# Paper-based isotachophoretic preconcentration technique for low-cost determination of glyphosate

**DOI:** 10.1007/s00216-024-05544-x

**Published:** 2024-10-01

**Authors:** Nicolás Franck, Pascal Stopper, Lukas Ude, Raul Urteaga, Pablo A. Kler, Carolin Huhn

**Affiliations:** 1 Centro de Investigación en Métodos Computacionales, UNL–CONICET, Predio CCT CONICET RN 168, S3000GLN Santa Fe, Argentina; 2 Instituto de Física del Litoral, UNL–CONICET, Güemes 3450, S3000GLN Santa Fe, Argentina; 3 Departamento de Ingeniería en Sistemas de Información, FRSF-UTN, Lavaise 610, S3000GLN Santa Fe, Argentina; 4 Chemistry Department, Eberhard Karls Universität, Auf der Morgenstelle 18, 72076 Tübingen, Germany

**Keywords:** Glyphosate, e-µPAD, Paper-based devices, Point-of-need devices, Isotachophoresis

## Abstract

Electrophoretic microfluidic paper-based analytical devices (e-µPADs) are promising for low-cost and portable technologies, but quantitative detection remains challenging. In this study, we develop a paper-based isotachophoretic preconcentration and separation method for the herbicide glyphosate as a model analyte. The device, consisting of two electrode chambers filled with leading and terminating electrolytes and a nitrocellulose strip as the separation carrier, was illuminated by a flat light source and operated with a voltage supply of 400 V. Detection was accomplished using a simple camera. Colorimetric detection was optimized through competitive complexation between glyphosate, copper ions, and pyrocatechol violet as a dye. The buffer system was optimized using simulations, (i) ensuring the pH was optimal for the demetallation of the blue pyrocatechol violet-copper complex [PV] to the yellow free dye and (ii) ensuring the electrophoretic migration of glyphosate into the slower [PV] for the colorimetric reaction. A new data evaluation method is presented, analyzing the RGB channel intensities. The linear range was between 0.8 and 25 µM, with a LOD of approximately 0*.*8 µM. The ITP separation preconcentrated glyphosate by a factor of 820 in numerical simulations. The method may be applied to control glyphosate formulations, especially in developing countries where herbicide sales and applications are poorly regulated.

**Graphical Abstract:**

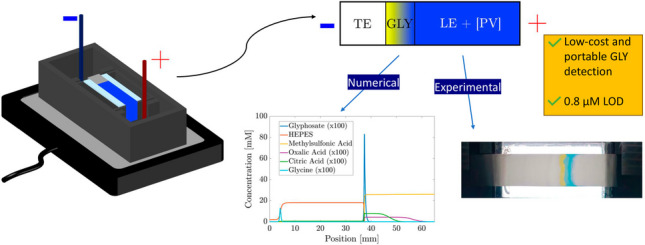

**Supplementary Information:**

The online version contains supplementary material available at 10.1007/s00216-024-05544-x.

## Introduction

Glyphosate (GLY) is the world’s most heavily applied herbicide. Usage worldwide has increased by a factor of 260 since its introduction in the agricultural market in 1974 [1]. GLY is widely used in agriculture, forestry, and urban applications [2]. Its overuse and abuse are discussed to evoke environmental problems [3]. Mobilization into surface water may pose a threat to ecosystems [4]; however, not all countries monitor GLY systematically [5]. For monitoring, liquid chromatography (LC) coupled to mass spectrometry (MS) after derivatization of GLY with 9-fluorenyl methyl carbonyl chloride (FMOC) is frequently used for quantification, making its analysis costly and time-consuming [6]. Alternative methods have been published based on spectroscopic methods, electrochemical sensors, or capillary electrophoresis [7]. Other analytical methods with lower demand for instrumentation have also been published, the most relevant ones are colorimetric methods. These methods reached limits of detection compatible with environmental monitoring, e.g., by using a fluorescent probe and competitive complexation with copper, requiring only a UV lamp as equipment [8]. Furthermore, a colorimetric method with naked eye detection was recently reported, where the complexation of Cu^2+^ with pyrocatechol violet (PV) leads to a blue complex. In contrast, GLY has a higher complex formation constant to Cu^2+^ than PV; thus, upon competitive complexation, GLY induces a shift in absorption wavelength upon forming the yellow uncomplexed PV. A limit of detection of 20 µM GLY in tap water by naked eye was demonstrated, and quantification by smartphone analysis with a limit of detection as low as 2*.*66 µM [9] was reported. Two main constraints are related to these approaches: first, surface waters have concentrations often below 6 nM [10, 11]; second, matrix effects, i.e., the presence of other complexing agents such as EDTA, which interfere by complexing copper with a higher complex formation constant than PV, will impair GLY analysis. To improve the limits of detection of these colorimetric techniques, sample preconcentration and matrix removal have to be implemented before the colorimetric detection of GLY. In this study, we examine the applicability of paper-based isotachophoresis for both tasks: sample preconcentration and electrophoretic removal of matrix compounds.

Paper-based colorimetric assays have been applied for more than 100 years, but more recently, microfluidic paper-based analytical devices have developed rapidly because of their advantages, such as small sample volume, rapid detection rates, low cost, and portability. They can be coupled with various detection methods including colorimetric, electrochemical [12], or optical detection, with simple detectors such as scanners, cameras in mobile phones, or more specialized devices [13]. The images taken can be analyzed using computer programs, and, depending on the color and hue of the image, the total value or just one channel of the color space can be used for quantification [14].

Most paper-based methods rely on capillary imbibition or diffusion phenomena to bring the sample into contact with the reactants of the specific colorimetric detection. However, these transport modes do not separate the analyte of interest from disturbing matrix compounds. This can be achieved using principles of active mobilization by using electric fields for charged compounds such as GLY.

Particularly, electrophoretic microfluidic paper-based analytical devices (e-µPADs) became attractive in recent years for analytical applications [15], often with the integration of smartphones and mobile devices for detection [16, 17]. For example, different types of human and bovine serum proteins were preconcentrated and separated quickly and with good resolution [18]. Biomarkers such as lipids, small molecules, carbohydrates, nucleic acids, and cells [19] were analyzed using e-µPADs. e-µPADs have also been studied to improve the limit of detection (LOD) of lateral flow immunoassays in diagnostic devices. High preconcentration factors of up to 900 were achieved by using isotachophoresis (ITP) [20], an electrophoretic method based on electrolyte systems with concentration gradients or discontinuous concentrations profiles. ITP was successfully integrated into microfluidics and paper-based chemical and biochemical assays [21]. ITP can concentrate trace amounts of substances into a narrow boundary region which improves limits of detection as shown in various applications [22]. Also, ITP is versatile in analyzing virtually any ionogenic analyte, making it broadly applicable in many analytical applications, especially in biomedical and environmental analyses [23].

This work aims to develop a simple, low-cost paper-based isotachophoretic device for GLY detection. Additionally, we seek to address current limitations in sensitivity for environmental monitoring and explore potential applications in developing countries where herbicide regulation is lacking.

## Materials and methods

### Chemicals

Glyphosate standard (analytical grade, 98.1%) was purchased from Merck, Darmstadt, Germany. The pyrocatechol violet dye was bought from Carl Roth, Karlsruhe, Germany, and copper(II) chloride dihydrate was delivered by Sigma-Aldrich, Steinheim, Germany. 2-(*N*-Morpholino)ethanesulfonic acid (MES, anhydrous, high purity grade) was purchased from Amresco (Darmstadt, Germany). HEPES (Lobov Cientifica, Buenos Aires, Argentina) was used to prepare background electrolytes (BGE) and terminating electrolytes (TE). HCl (Laboratorios Cicarelli, San Lorenzo, Argentina) was used as the leading electrolyte (LE); *ε*-aminocaproic acid and TRIS (Merck, Darmstadt, Germany) were used as counter-ions (CI). Polyvinylpyrrolidone (MW 1,000,000) was purchased from Polysciences, Warrington, USA. All solutions were prepared with ultrapure water from an inverse osmosis purifier (Osmoion, Apema SRL, Villa Dominico, Argentina). The pH of solutions was set by modeling the concentrations using pK_a_ values and the pH was confirmed with a pH meter (Adwa A12, Szeged, Hungary). The chemicals used for the BGE in CE-MS analysis were aqueous ammonia solution (LC–MS grade, 25%) and formic acid (FA) for LC–MS (98%). Aqueous HCl (32%) for preconditioning was purchased from Thermo Fisher Scientific (Schwerte, Germany). Isopropanol (LC–MS grade) and phosphonic acid (H_3_PO_3_) (99%) were purchased from Merck, Darmstadt, Germany. All chemicals were used without further purification. The water (H_2_O) used as solvent was deionized and purified by an ELGA LabWater water purification system (Celle, Germany).

### Optimization of the colorimetric detection

The spectral analyses were carried out using a Lambda 19 UV/Vis spectrometer (PerkinElmer, Waltham, MA, USA) with LambdaSPX software (Version 1.7, ascanis OHG, Überlingen, Germany) and UV transparent semi-micro cuvettes, width: 10 mm (UV-Polymer, Brand, Wertheim, Germany). Cu(II)-GLY ([GLP]) stability constants were found to be 11.8 [24] and 11.9 [25, 26]. For the copper-PV complexes ([PV]), no stability constant was found in the literature. Still, results by Yadav and Zelder indicate a similar stability constant as for [ZnZCN] at pH 7.4 based on its sensitivity for GLY [9], with a reported stability constant at pH 7.4 of 5.7 [27]. It is important to note that the stability constants of the complexes depend on pH, temperature, ionic strength of the solution, and concentration of the metal ions and ligands, so these log(*β*) values do not always match the conditions of the assay but can be used as an estimate of a cutoff value for competitive complexation.

#### Testing porous substrates

Different substrates for e-µPADs were investigated: three solid supports were used in the experiments: cellulose filter paper MN615 (Macherey–Nagel, Düren, Germany), Nitrocellulose Hi-Flow HFC13504 (Merck, Darmstadt, Germany), and ALUGRAM RP-18W/UV_254_ (Macherey–Nagel, Düren, Germany). To investigate the applicability of different porous substrates for electrophoretic experiments with colorimetric detection, sheets of 5 mm × 5 mm in size were cut from the solid supports. Aqueous solutions of [PV] at a concentration of 75 µM were prepared by mixing the pyrocatechol violet dye and copper chloride at a stoichiometric ratio of 1:2 in solutions of MES (10 mM) adjusted to pH 6.5 with KOH. Aqueous GLY solutions with a concentration range of 0–500 µM were tested. Six sheets were prepared for each substrate.

After some optimization steps, the test procedure added 20 µL of the GLY samples with five different concentrations to five sheets. The sixth sheet was used as blank. The papers were allowed to dry at ambient temperature. Then, 10 µL of the colored complex were added. Photographs were taken at each stage.

### CE-MS analysis

ITP-UV analysis was conducted with an A 7100 CE instrument from Agilent Technologies (Waldbronn, Germany). The separations were performed in a bare fused silica capillary with 50 µm inner diameter and 75 cm in length. Before the first use, capillaries were conditioned by flushing the surface with 0*.*05 M aqueous NaOH and H_2_O for 180 s, each at 1 bar. Before ITP measurements, the capillary was dynamically coated by flushing the surface with 90 mM aqueous polyvinylpyrrolidone (PVP) solution for 600 s at 1 bar. Separations were conducted with an internal pressure support of 40 mbar and a voltage of −10 kV. For detection, the built-in diode array detector was used at a wavelength of 596 nm for the [PV] complex and 444 nm for free PV.

CE-MS analyses were conducted with a 7100 CE coupled to a 6150 single quadrupole mass spectrometer from Agilent Technologies (Waldbronn, Germany) via a coaxial sheath liquid electrospray interface equipped with a platinum needle. The sheath liquid consisted of a mix of 50% H_2_O and 50% isopropanol with 0.1% formic acid added. The sheath liquid was supplied via a splitter by a 1200 series isocratic pump from Agilent Technologies (Waldbronn, Germany) at a flow rate of 5 µL*/*min. The drying gas temperature was 150 °C, with a gas flow of 11 L*/*min and a nebulizer pressure of 3 psi. The MS was operated in selected ion monitoring mode, with 100 V fragmentor voltage and a capillary voltage of 4000 V. Molecular ions monitored were [M-H]^*−*^. The separations were performed in a bare fused silica capillary with 50 µm inner diameter and 75 cm in length. Before the first use, capillaries were conditioned by flushing the surface with 0*.*05 M aqueous NaOH and H_2_O for 180 s each at 1 bar. The separations were conducted at an internal pressure support of 50 mbar and a voltage of −15 kV increasing over 2 min to −30 kV after injection. The injection was conducted via hydrodynamic injection at 50 mbar for 10 s, followed by an injection of background electrolyte at 100 mbar for 10 s.

### Computational tools

Numerical simulations were performed using OpenFOAM v2212. Meshing processes were made with blockMesh integrated into the solver. The number of cells for all cases was kept approximately constant in the range of 15,000 with a uniform spatial distribution. The solver selected within the OpenFOAM environment was the electroMicroTransport solver for electrophoretic separations [28]. All the simulated cases were run on an AMD Ryzen 5 3400G 3.70 GHz (1 CPU, 4 cores), 16 GB Micron 16 GB-DDR3-1600 MHz. A Whatman #1 model was used for the simulations. electroMicroTransport used two parameters: the porosity, measured by weighting dry and wet substrates with known geometries; and the tortuosity (*τ*), related to the constriction factor *C* by *C* = *τ*^2^. This factor was calculated using the experimental setup of the electrical model [29]. The simulation geometry was a rectangular paper strip, originally of 80 mm length and 10 mm width, but with an effective electric field application distance of 65 mm. Electrolyte electrophoretic mobilities were taken from PeakMaster, except GLY taken from previous CE measurements [30]. Initial conditions included the TE + GLY located 18 mm from the TE reservoir, with the remainder filled with LE. The solver considers the reagent species’ electrophoretic transport and a null electroosmotic flow (EOF). Boundary conditions for LE and TE were set to no flux anywhere. Dirichlet boundary conditions at the reservoirs imposed a prescribed potential difference for the electric potential.

### Paper-based electrophoresis

**Fig. 1:**
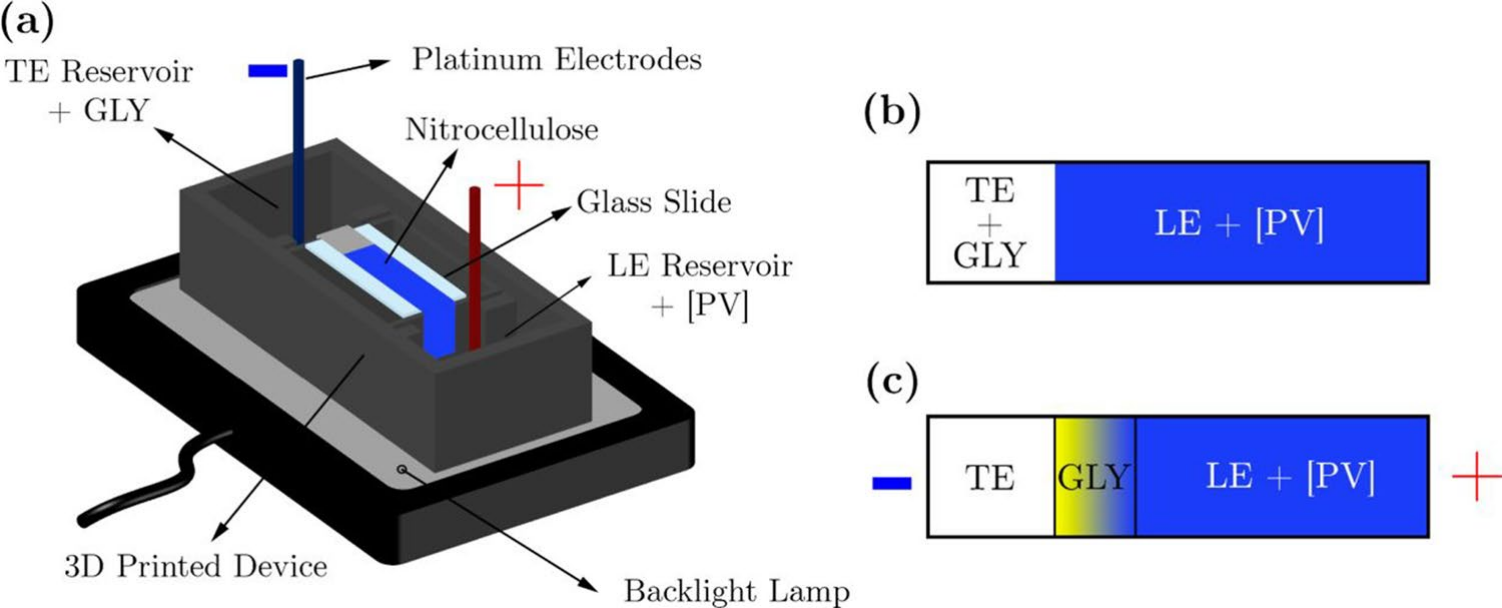
(**a**) Schematic of the low-cost e-µPAD device, featuring a 3D-printed housing with platinum electrodes and nitrocellulose strips bridging the anode and cathode reservoirs. (**b**) Configuration after filling reservoirs and before starting electrophoretic migration. (**c**) Glyphosate preconcentration and color change upon applying an electric field for some minutes

A new low-cost device for e-µPAD was developed and is schematically shown in Fig. 1a. It consists of a 3D-printed housing with a separation chamber with anode and cathode reservoirs, each equipped with platinum electrodes. A nitrocellulose strip of 80 mm length and 10 mm width was cut with a paper cutter and placed on a glass support bridging the two reservoirs with its ends being immersed in the buffers. A flat backlight lamp (Rollei Lumis Key Light LED streaming light, Rollei, Germany) was used for illumination.

#### Paper-based capillary electrophoresis

Based on our results for the colorimetric analysis reaction detection, we developed a paper-based capillary electrophoresis (pCE), using MES as a BGE adjusted to pH 6.5 with KOH. For the colorimetric complex solution, 175 µM of PV was mixed with 2 equivalents of CuCl_2_ salt in the BGE buffer solution. GLY solutions at different concentrations (100, 150, 400, and 1200 µM) were prepared in the background electrolyte. In all electrolyte solutions, 3% m/v of PVP were added to suppress the EOF.

#### Paper-based ITP

Isotachophoresis experiments used 2*.*5 mM aqueous HEPES solution as TE with 5 mM *ε*-aminocaproic acid as counter-ion; 26 mM aqueous HCl solution was used as the LE with a mixture of *ε*-aminocaproic acid and TRIS (26 mM each) used as a CI. All electrolytes were chosen and optimized to ensure a pH value of 6.5 optimal for the colorimetric detection, using PeakMaster [31] and numerical simulations, as described in the “Computational tools” section. In the protocol for ITP experiments, hydrophobic double-sided adhesive tape was placed at the ends of the glass slide to fix the nitrocellulose strips and keep an air gap between the glass avoiding edge effects that could alter imbibition flow [32]. The anode reservoir was initially filled with a solution of the [PV] colorimetric complex at a constant concentration of 175 µM in the LE. After 150 s, the cathode reservoir was filled with GLY solutions dissolved in the TE. During the imbibition process, GLY and the [PV] complex moved towards each other through diffusional transport, eventually achieving the configuration displayed in Fig. 1b.

Once the nitrocellulose sheet is completely wetted, ions start electrophoretic migration upon application of the electric field. This configuration leads to a semi-infinite sample loading of GLY and thus a continuous sample focusing by ITP (i.e., the amount of sample focused in an ITP stack increases with time) [21]. The color change occurs when preconcentrated GLY is separated from the TE, and reaches the [PV] complex, which has a lower electrophoretic mobility (see Fig. 1c), as determined by CE-UV (see the “Verification of the separation by ITP-UV analysis” section). A 400 V electric potential is provided by a picoammeter/voltage source (Keithley 6487, Cleveland, OH, USA), which also measures the applied current. Color changes are recorded on a high-resolution digital camera (Canon EOS 1000D), placed vertically at a distance of 30 cm, connected to a PC, and set to burst mode. The spatial resolution was 36 µm and the time resolution was 12 frames per minute. Images obtained from experiments are analyzed with an ad hoc algorithm developed in the MATLAB software framework.

## Results and discussion

### Optimization of the colorimetric detection

**Fig. 2:**
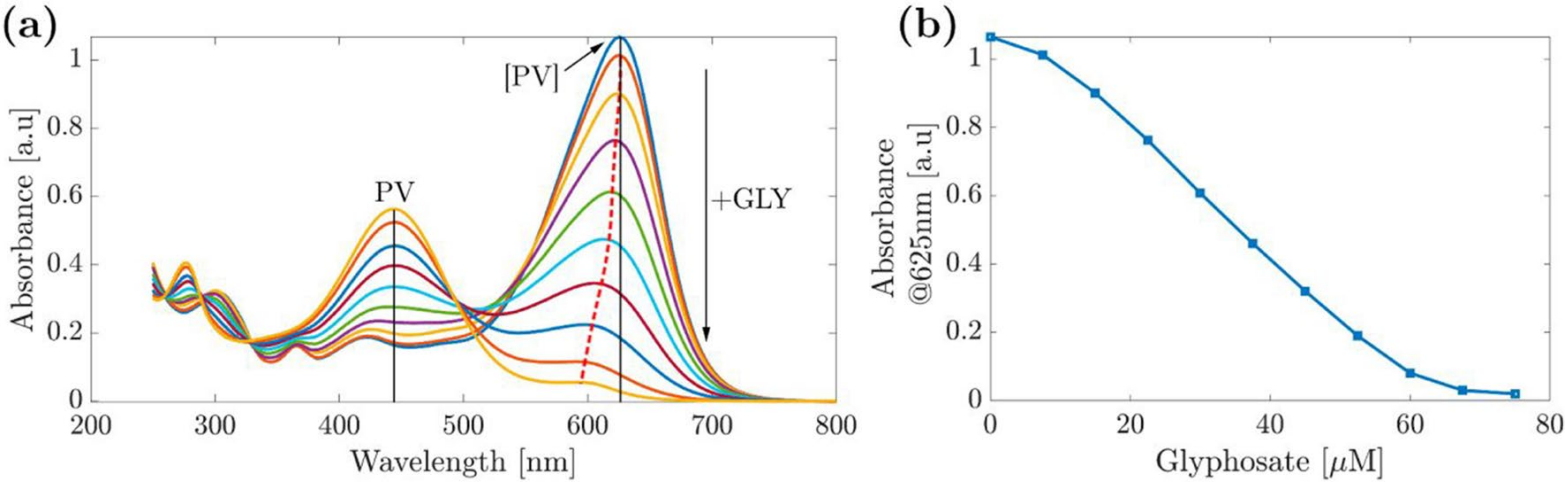
Colorimetric GLY analysis in solution: (**a**) UV/Vis spectra of PV (30 µM) in the presence of two equivalents Cu^2+^ at pH 6.5 (aqueous MES buffer) with increasing concentration of GLY (**b**) absorbance at 625 nm in MES buffer at pH 6.5 depending on the GLY concentration

The colorimetric detection was based on the work of Yadav and Zelder [9]. Pyrocatechol violet forms complexes with copper. Upon the addition of GLY, which has a higher complex formation constant with Cu^2+^ than PV, the [PV] complex dissociated and changed color from blue to yellow, indicating the formation of free PV. Yadav and Zelder already tested many analytical parameters of the reaction of GLY with the [PV] complex. In the final protocol, the authors used a pH of 6.5 in HEPES buffer to be suitable for GLY detection. However, as HEPES with a pK_a_ of 7.41 at 20 °C [33] is not well suited as a buffer for pH 6.5, we preferred MES (pK_a_ ≃ 6.15 at 20 °C) [34]. To minimize risks of complex decomposition, we stored aqueous solutions of the reaction partners PV, CuCl_2_, and MES separately at 4 °C. The [PV] complex in MES buffer (pH 6.5) was prepared directly before it was applied for GLY analysis upon mixing these solutions. Concentration series with increasing equivalents of GLY were conducted for the complexes in the MES buffer to obtain calibration curves, define the linear range, and calculate the LOD. As depicted in Fig. 2a, the light blue color of [PV] (absorbance maximum at 625 nm) with a stoichiometry of PV:Cu^2+^ of 1:2 turns yellow upon the addition of GLY, as added GLY competes with [PV] for the Cu^2+^ ions.

Copper complexation by GLY thus releases free PV (absorbance maximum at 444 nm). When using 30 µM of the copper [PV] complex (2 equivalents Cu^2+^), the calibration curve in Fig. 2b reveals a linear range from 1 to 60 µM. The limit of detection was ∼1 µM using the lower end of the linear range for UV/Vis analyses and ∼15 µM for naked eye detection.

#### Testing porous substrates

**Fig. 3:**
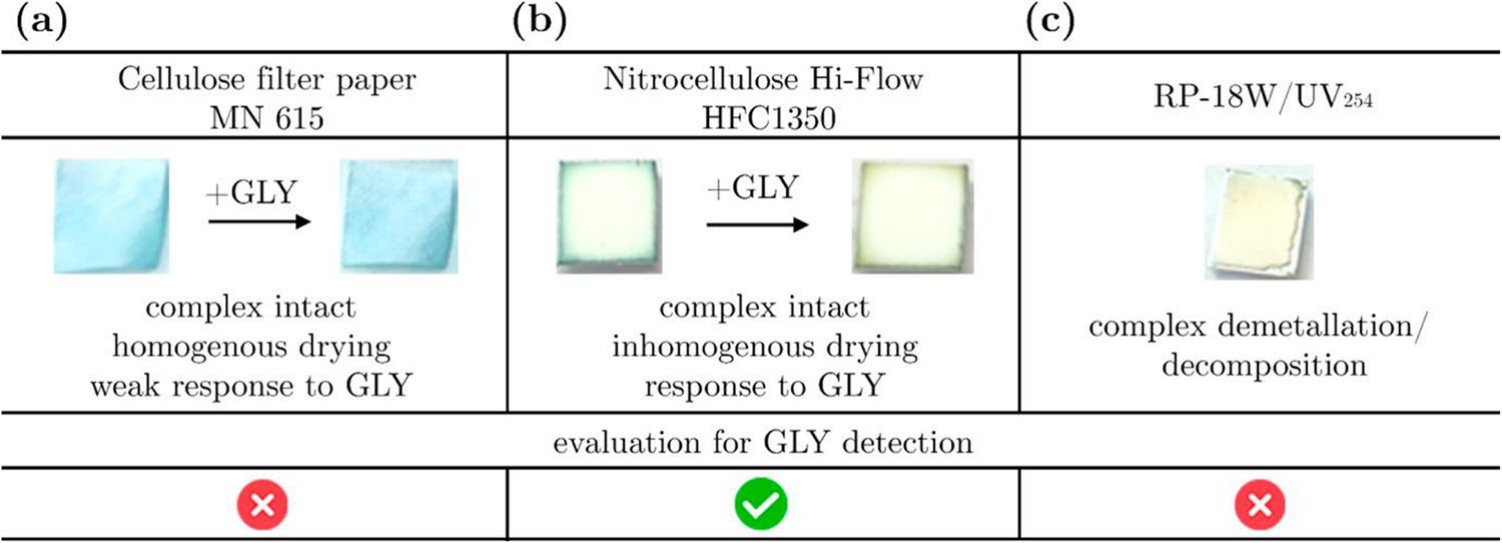
Investigation of solid supports after applying solutions of [PV] complex and GLY. (**a**) Cellulose filter paper did not show a visible color change at high GLY concentrations. (**b**) Nitrocellulose shows a color change but suffers from the coffee ring effect. (**c**) The yellow color of RP-18 W TLC plates after the addition of [PV] indicates the decomposition of [PV] complexes

The transfer of the batch method for colorimetric detection faced three major challenges: sorption of reaction partners, especially GLY on the solid substrate; complex dissolution; and strong diffusional broadening of spots. Therefore, three solid supports were chosen providing orthogonal surface chemistries. In regular filter paper, composed of hydrophilic cellulose (cellulose filter paper MN 615), [PV] complex solutions spread evenly (see Fig. 3a), and the blue color remained, which indicated that the complex remained intact. However, when adding GLY solution at high concentrations, no visible color change from blue to yellow occurred, as observed in Fig. 3a. We hypothesize that, despite the high stability constant of the [GLP] complex, a strong interaction of GLY with the cellulose substrate was present as can be expected from GLY’s interaction with –OH and O^*−*^ groups, simulated in theoretical studies [30, 35] and well known from its sorption to soil mineral oxides [36].

Using nitrocellulose, we expected GLY adsorption onto the solid support to be reduced by electrostatic repulsion. Looking at the photographs in Fig. 3b, [PV] complexes stayed intact on the nitrocellulose substrate and a color change from blue to yellow occurred when GLY was added. However, the interaction of copper complexes ([PV] and [GLP]) and free GLY with nitrocellulose was lowered to such an extent that the components diffused to the edges of the sheets upon drying. This phenomenon, named the coffee ring effect, is due to the solvent evaporating faster in the middle of the strip, evoking a concentration gradient [13]. Moreover, the EOF in nitrocellulose is considerably higher than the EOF in other paper substrates [37], which generates distortions in the electrical profile of electrophoretic separations. Consequently, PVP was added to buffer solutions to suppress such flow. For the “RP-18 W” TLC plates, the surface was well wetted with aqueous solutions of the complexes due to residual –OH groups of the SiO_2_ particles, but decomposition or demetallation of [PV] complexes can be deduced from the loss of the blue colors upon drying the [PV] solution and the formation of the free yellow PV colorant, as can be seen in Fig. 3c. We proceeded with nitrocellulose as a solid support for paper-based electrophoresis and ITP.

### Numerical electrolyte optimization for ITP

To identify the most suitable buffers for paper-based ITP experiments, we conducted numerical simulations using a system with three different counter-ions and three different terminating electrolytes. The primary factors considered were (i) optimal pH for the colorimetric reaction of [GLP], (ii) preconcentration factors reached for GLY in the ITP system, (iii) electromigration behavior of GLY and [PV] to assure that competitive complexation takes place, and (iv) influence of the buffer components on the stability of the [PV] complex.

Given the challenges involved in modeling nitrocellulose particularly due to the lack of data on its tortuosity and the effects of PVP on suppressing the EOF, we utilized a Whatman #1 paper model for the numerical experiments [38]. The TE and LE electrolyte concentrations were derived from the work of Moghadam et al., who explored the integration of ITP onto nitrocellulose-based paper microfluidic devices to enhance the detection limits of lateral flow immunoassays and reported 900-fold increase in initial sample concentration [20]. For all simulations, we used 26 mM HCl as LE and 5 mM *ε*-aminocaproic acid as the CI in TE. Glyphosate sample concentration was set to 1 µM, and pH calculations were done numerically [39].

**Fig. 4:**
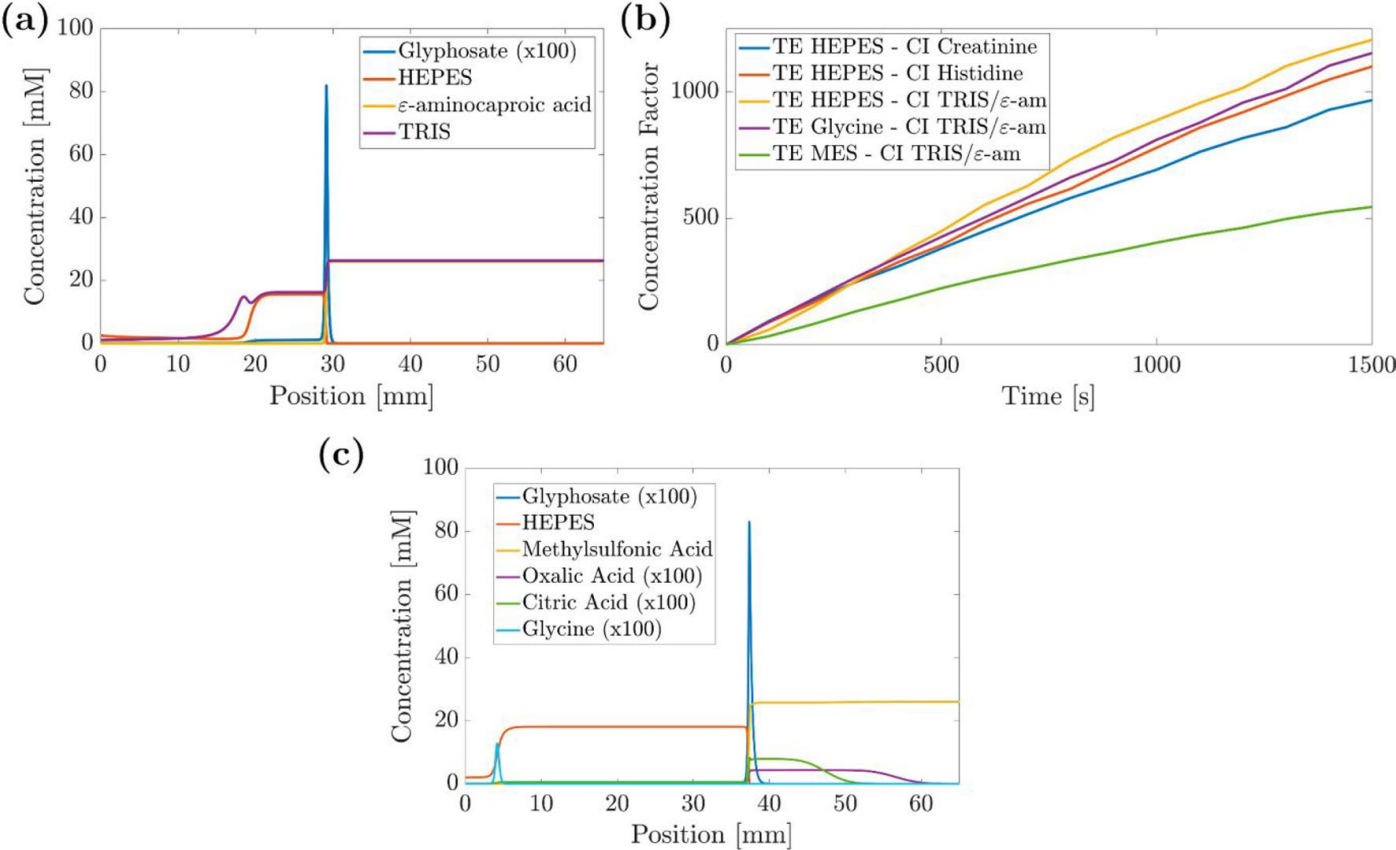
Results of numerical simulations for optimizing electrolyte combinations in paper-based ITP. (**a**) Glyphosate sample and electrolyte arrangement (Combination 3 in Table 1) at 900 s for counter-ions TRIS and *ε*-aminocaproic acid and TE HEPES. (**b**) Preconcentration factors for different combinations of counter-ions and terminating electrolytes (see Table 1) depending on the time used for preconcentration. (**c**) Matrix effects with glycine, oxalic acid, and citric acid. HCl in the LE was replaced by 26 mM methylsulfonic acid

Exemplarily, Fig. 4a shows the results for GLY preconcentration after 900 s in an ITP using electrolytes of combination 3: LE: HCl, TE: HEPES with the counter-ions TRIS and *ε*-aminocaproic acid. Glyphosate is well preconcentrated from 1 to ∼820 µM behind chloride as the LE into a sharp peak at a position of 30 mm. From this, we calculate a preconcentration factor of 820. Between 20 and 30 mm, the GLY concentration is ∼11 µM, not yet fully adapted. Using longer separation times, higher concentrations are reached. The dependence of the preconcentration factor on the simulation time is shown in Fig. 4b for all buffer combinations tested. Similar slopes and thus similar preconcentration factors were observed for combinations 1–4 with glycine and HEPES as TE. Electrolyte combination 5 was inferior in preconcentration and the limited performance with MES as the TE is evident. However, further aspects have to be considered for GLY analysis: for instance, using creatinine as a CI did not meet the pH requirements of the colorimetric detection (pH range 6*.*5–7*.*4) with a pH of 5 in the LE solution. Additionally, when we experimentally tested histidine as CI and glycine as TE, a strong interaction with the [PV] was observed, resulting in a color change before the addition of GLY when the zone of the [PV] and TE met (data not shown). Thus, further optimization was focused on electrolyte combination 3. In environmental samples, we may expect other complexing agents such as oxalic acid or citric acid, which we know from colorimetric batch testing to disturb the glyphosate analysis. While compounds such as glycine and other ampholytes are not included in the ITP stack due to too low mobilities, oxalic acid and citric acid would disturb the analysis with the buffer system chosen. We thus conducted further simulations and found methylsulfonic acids as a suitable LE ion for samples with complexing agents as matrix constituents. Figure 4c shows the results of the simulation demonstrating that glycine is not included in the ITP stack and oxalic acid and citric acid are well separated from glyphosate. However, it is important to note that with methylsulfonic as LE, the theoretical preconcentration factor is slightly smaller than when using HCl. For this reason, we chose HCl as LE for the experimental work. The buffer system based on methylsulfonic acid can be implemented in the future for complex matrices without modifying the experimental setup.

**Table 1:** Electrolyte combinations used in numerical simulations, displaying the concentrations, pH values, and preconcentration factors (*C*/*C*_i_), calculated as the ratio between GLY concentration at 900 s (C) and the initial concentration (*C*_i_)

Combination	TE [mM]	CI (LE) [mM]	LE pH	*C*/*C*_i_ (*t* = 900 s)
1	HEPES — 2.5	Creatinine — 60	5	635
2	HEPES — 2.5	Histidine — 60	6.2	700
3	HEPES — 2.5	TRIS/*ε*-aminocaproic acid — 26	6.5	820
4	Glycine — 2.5	TRIS/*ε*-aminocaproic acid — 26	6.5	725
5	MES — 2.5	TRIS/*ε*-aminocaproic acid — 26	6.5	365

### Verification of the separation by ITP-UV analysis

To verify the relative migration of GLY, [PV], and the free PV, the system established in the paper-based ITP was adapted to be reproduced in a classical capillary electrophoresis setup. For this, PVP in the electrolytes was substituted by a dynamic PVP coating [40] applied to the capillary surface before measurements. Additionally, the separation voltage was set to −10 kV with a constant pressure support of 40 mbar for mobilization. To start the measurement, the capillary was initially flushed with LE consisting of the buffer system with the [PV] complex. Subsequently, the TE was introduced by the pressure support, and a voltage was applied, leading to the formation of an ITP in the capillary.

**Fig. 5:**
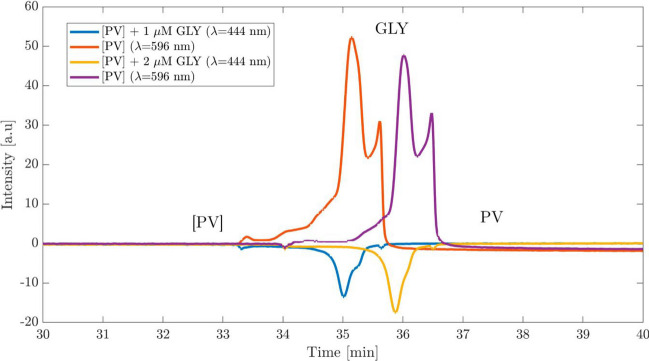
ITP-UV measurement of [PV] (596 nm) and free PV (444 nm) in a commercial CE instrument. Measurements were conducted in a capillary with 50 µm inner diameter at a separation voltage of −10 kV and 40 mbar with a LE consisting of 26 mM *ε*-aminocaproic acid, 26 mM HCl, 26*.*3 mM TRIS and 175 µM [PV]. The TE consisted of 2*.*5 mM HEPES, 5 mM *ε*-aminocaproic acid, and 1 and 2 µM GLY

The ITP-UV electropherograms displayed in Fig. 5 show the successful reproduction of the ITP for 1 and 2 µM GLY in the TE when using ITP-UV analysis. GLY was detected indirectly by the UV absorption of free PV after dismantling the [PV] in the presence of GLY [9]. For detection, the absorption maximum of the complex 596 nm was chosen while the free PV was detected at 444 nm. A preconcentration of GLY from the TE can be seen in front of the ITP stack although GLY is constantly preconcentrated from the TE. The separation was susceptible to varying EOF due to the dynamic coating but no attempts were made for optimization. The steady decline of the baseline intensity is associated with the low stability of the complex in the buffer medium. A reduction in the signal can be seen at both observed wavelengths in the electropherograms as impurities are also preconcentrated in the ITP stack before GLY.

### Paper-based electrophoresis

#### Paper-based capillary electrophoresis

**Fig. 6:**
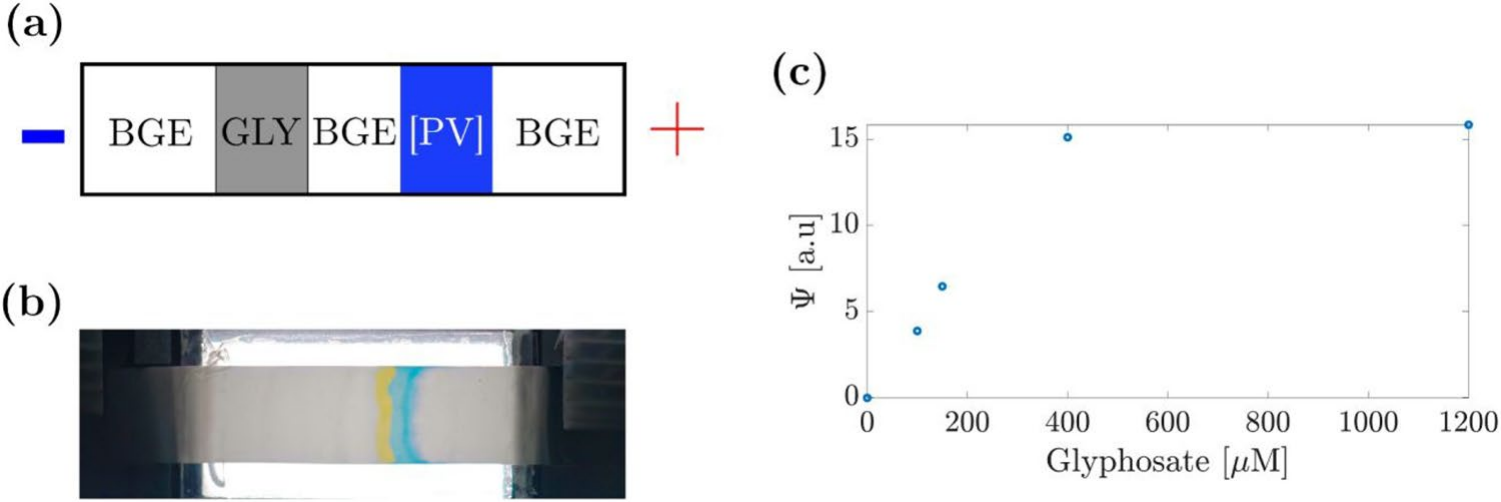
Initial setup and results of paper-based capillary electrophoresis (pCE) using MES buffer (pH 6.5) and PV complex for GLY detection. (**a**) Configuration of the nitrocellulose strip with [PV], BGE, and GLY solutions before voltage application. (**b**) Experiment photograph at *t* = 8 min upon electric field application, with GLY having reached the [PV] band. (**c**) Absorbance changes indicating GLY concentration with a detection range from 100 to 400 µM

The experimental protocol consisted of first placing a nitrocellulose strip on the glass support bridging the two reservoirs. We added 20 µL of [PV] solution to the right of an imaginary line in the middle of the nitrocellulose strip. Then, further bands were added to the left: 10 µL BGE and 20 µL GLY (see scheme in Fig. 6a). Afterwards, both cathode and anode reservoirs were filled with BGE solutions. Upon electric field application (1500 V m^*−*1^), GLY will migrate to the cathode with a higher velocity than [PV], reaching the colorimetric complex band which evoked the formation of the yellow free dye and the glyphosate-copper complex (see Fig. 6b).

The calibration curve (Fig. 6c) showed a saturation starting at about 400 µM of GLY forming the upper end of the linear range. Differences to the 1200 µM sample are negligible, so the optimal detection range for pCE was 100–400 µM of GLY when using a starting concentration of 175 µM of [PV]. Image analysis was identical to the ITP experiments, described in the “Paper-based ITP” section. Lower GLY concentrations were tested, but already 50 µM was below the LOD. We estimated the LOD to be ∼100 µM, not suitable for environmental samples.

#### Paper-based ITP

**Fig. 7:**
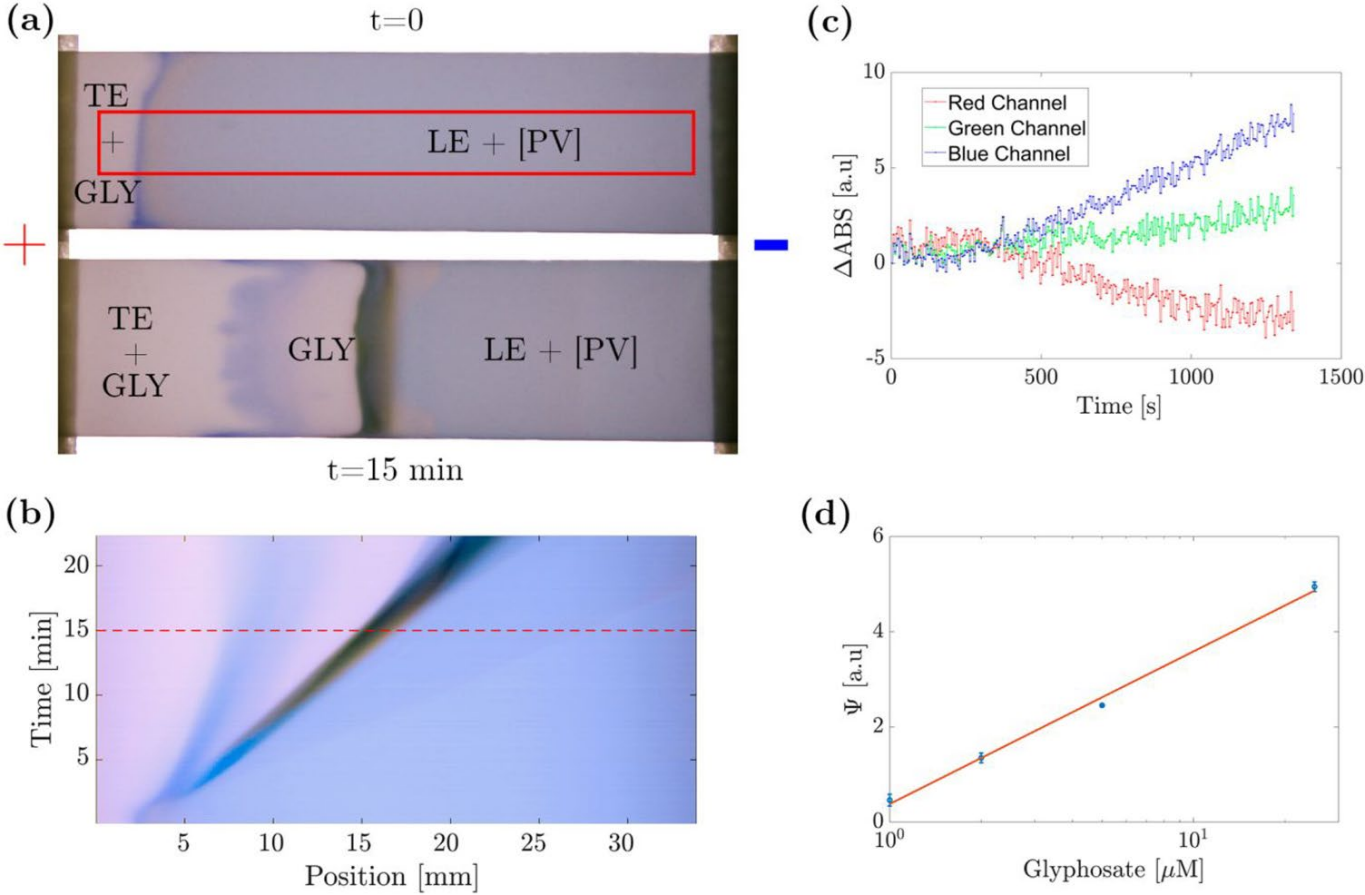
Results of GLY detection with an e-µPAD by ITP technique with colorimetric detection. (**a**) Initial and 15-min photographs with the analysis area marked in red. (**b**) Time–space matrix showing the system’s dynamics. The dotted line contains information from the vertically averaged photograph (a), at *t* = 15 min. **(c)** ∆*ABS* values integrated along the spatial coordinate, for each channel over time. (**d**) Calibration curve for multiple GLY concentrations (1, 2, 5, and 25 µM) of integrated absorption difference index (*Ψ*)

Using the pCE technique, a high LOD of 100 µM for GLY was reached while the LOD of batch experiments was 2*.*66 µM for smartphone detection [9] and 1 µM with our high-end UV/VIS spectrometer. Thus, the pCE technique was replaced by paper-based ITP to enable preconcentration. For ITP experiments, we used four different GLY concentrations (1, 2, 5, and 25 µM) along with a plain buffer as a blank. Each experiment was repeated at least twice. Electrolyte combination 3 (see Table 1) was used as the electrolyte system. The nitrocellulose was prepared as described in the “Paper-based ITP” section. To mitigate the EOF, 3% m/v of PVP were added to TE and LE solutions. We chose a rectangular area in the photograph to process the images taken during each experiment. Its size was set to the length of the nitrocellulose strip and one-third of its width (34 mm × 3*.*3 mm) as shown in Fig. 7a at time 0 of an experiment with a GLY concentration of 5 µM. Figure 7a shows the LE containing the blue [PV] complex before applying voltage. A sharp blue zone is visible on the left, where the TE with GLY added meets the [PV], due to the coffee ring effect during the capillary imbibition of the strip. The effective preconcentration of GLY by ITP was well visible in the second image of Fig. 7a, after 15 min time of separation applying a voltage of 400 V. A video summary of this entire experimental run is linked in the supplementary information. After 15 min upon applying the electric field, intense colors are visible in the center of the nitrocellulose strip. As indicated in Fig. 1c, different zones can be expected (discussing only the anions): (i) a zone of GLY in TE, (ii) a zone with a mixture of the [GLP] complex and free PV, and (iii) a zone of [PV] in the LE. Due to continuous separation and the mixing of blue and yellow compounds, different mixed colors evolve, making it difficult to achieve a direct naked eye evaluation of the results.

For a complete analysis of the images, we constructed time–space matrices for all (RGB) channel intensities. For this, a vertical average of the images in the rectangular analysis area was calculated at each time point and all lines and stacked over time (Fig. 7b). Thus, the length coordinate is represented on one axis, while time is represented on the other, as depicted in Fig. 7b. This method visualizes the system’s dynamics of the entire experiment in a single image, showing the main colorimetric complexes involved.

The intensity of each channel (RGB) of the matrix can be written using Beer-Lambert’s law:

1 $${I}^{RGB}(x,t)={I}_{0}^{RGB}(x){e}^{-({\alpha }_{0}+{\alpha }_{[PV]})d}$$

where ${I}_{0}^{RGB}(x)$ represents the intensity of the backlight lamp. *α*_0_ and *α*_[*PV*]_ are the porous substrate’s and [PV]’s absorption coefficients, respectively, while *d* is the thickness of the porous layer. The absorption coefficient of the colorimetric complex can be linearly related to its respective concentration, i.e., *α*_[*PV*]_*d* = *β*^*RGB*^[*C*]_[*PV*]_(*t*), where *β*^*RGB*^ represents a variable that depends on the wavelength of the incident light, as measured spectra suggest (see Fig. 2a) and [*C*]_[*PV*]_(*t*) is the concentration of the [PV] at time *t*. Applying logarithm and separating the terms yields:

2 $$log(I(x, t)) = log({I}_{0}(x)) - {\alpha }_{0d} + {\beta }^{RGB}{[C]}_{[PV]}(t)$$

If the left term of the above equation for a photo at a given time *t* is subtracted from a photo at the initial time *t*_0_, we find an expression that eliminates the influence of the incident light and simplifies the terms related to nitrocellulose and only depends on the concentrations of the [PV] complex:

3 $${\Delta ABS}^{RGB}(x, t) = log(I(x, 0)) - log(I(x, t)) = {\beta }^{RGB}({[C]}_{[PV]}(t) - {[C]}_{[PV]}(0))$$

Given that the [PV] complex preferentially absorbs in the wavelength range corresponding to the red channel of the image and the uncomplexed PV preferentially absorbs in the blue channel (see Fig. 2a), ∆*ABS*^*R*^ for the red channel will be directly proportional to the amount of [PV], and ∆*ABS*^*B*^ for the blue channel to the concentration of PV. In the presence of GLY, we will thus observe an increase in ∆*ABS*^*B*^ and a decrease in ∆*ABS*^*R*^. In Fig. 7c, the values of ∆*ABS* for each channel integrated along the spatial coordinate are shown as a function of time for a GLY concentration of 5 µM. It can be seen that after an initial period, ∆*ABS*^*R*^ increases linearly, while ∆*ABS*^*B*^ decreases consistently. Additionally, it can be noted that the green channel does not show significant changes, which is consistent with the fact that the PV present in the experiment has similar absorption in that wavelength range (see Fig. 2a). Considering the above, a calibration curve was constructed from an integrated absorption difference index (*Ψ*), which integrates the difference between absorbance deltas between 0 and *t*_*ref*_: $\Psi ={\int }_{0}^{{t}_{ref}}$(∆*ABS*^*B*^ − ∆*ABS*^*R*^) *dt*, with a chosen *t*_*ref*_ of 900* s*. In Fig. 7d, this index is shown with a point for the average value of all measurements and an error bar for the standard deviation. A linear relationship between the logarithm of GLY concentrations and *Ψ* was adjusted: *y* = 1*.*24*x* + 0*.*38, *R*^2^ = 0*.*996. The LOD for this technique was 0*.*8 µM and the linear range of application was 0.8–25 µM.

#### Validation of the preconcentration

**Fig. 8:**
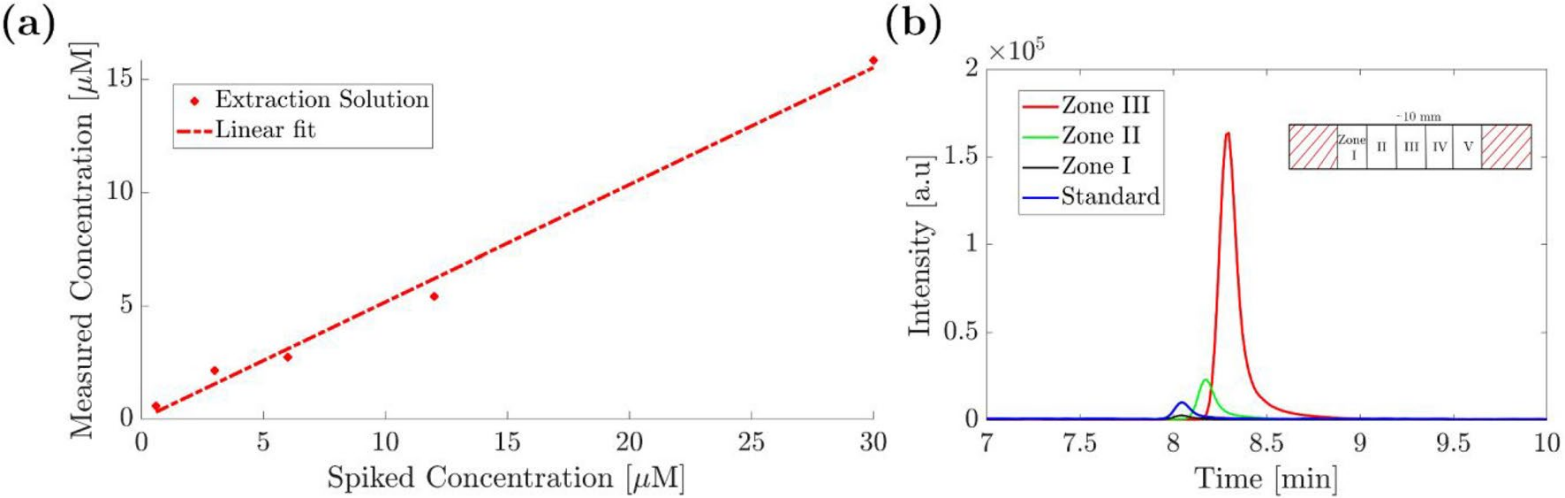
Results of the validation of the preconcentration: (**a**) calibration of the extraction solution with an efficiency of ∼ 50% (**b**) Nitrocellulose segments (zones) analyzed by CE-MS with a preconcentration factor of 180 for GLY in paper-based ITP

We validated the preconcentration factor of GLY in paper-based ITP in two steps. First, we performed a calibration for GLY concentrations on nitrocellulose. For this, 40 µL of an aqueous solution of GLY at concentrations of 0.6, 3, 6, 12, and 30 µM were dropped on sheets of nitrocellulose (10 mm × 10 mm) and the solution was allowed to dry at ambient conditions. Glyphosate was then extracted by inserting the nitrocellulose sheets into a microreaction vial with 250 µL of an aqueous solution of 37 mM H_3_PO_3_ and 112 mM NaOH. The extraction was accomplished under sonication for 1 h. Quantification was achieved using CE-MS (see the “CE-MS analysis” section) [41]. Extracted concentrations of GLY were determined using the isotope-labeled GLY standard glyphosate-2-^13^C (≥ 99.9%, Sigma-Aldrich, Steinheim Germany) added to the vial after extraction and before CE-MS measurements at a concentration of 6 µM. Comparing the concentrations of the GLY extracted from the nitrocellulose with the concentration of the internal standard, we see a slope of 0.5 for the regression line, indicating an extraction efficiency of about 50% (see Fig. 8a). Finally, a paper-based ITP experiment on nitrocellulose was conducted as before with a GLY concentration of 1 µM in the reservoir, but without using PVP to avoid impairments of extraction or downstream CE-MS analysis. After the ITP separation was complete, the paper strip was uniformly cut into 5 segments of about 10 mm length excluding the sections immersed in the reservoirs (see inset in Fig. 8b). Each segment was analyzed for GLY by CE-MS using the internal standard. Afterwards, the extracts for each paper segment and also the standard (in the same way as described above) were analyzed by CE-MS with the electropherograms shown in Fig. 8b. We estimated a preconcentration factor by comparison with the internal standard. For the zone in the center of the nitrocellulose strip, zone III, the highest peak area was obtained with a ratio of ∼15 between GLY and the internal standard indicating a concentration of (15 × 6) = 90 µM. With the extraction efficiency of 50%, a preconcentration factor of 180 was calculated for the starting concentration of GLY of 1 µM. This factor is not fully comparable to the one in the ITP experiment, as the effective preconcentration factor at the GLY front visible by colorimetric detection can be expected to reach considerably higher local values.

## Conclusions

Our study demonstrates that with a simple electrophoretic microfluidic paper-based device using ITP, preconcentration factors of up to 820 can be expected from simulation results and meet the requirements such as pH and effective electrophoretic mobilities of reaction partners in colorimetric detection. A preconcentration factor of about 180 was determined by offline analysis in segments of the nitrocellulose strips after ITP analysis. The instrumental setup was a simple 3D-printed device with two electrode chambers and filter paper; here, we used nitrocellulose to reduce sorption of the analyte GLY and decomposition of the colorimetric complex of pyrocatechol violet and copper ions. The electrophoretic mobilities and starting conditions were chosen to have preconcentrated GLY in the terminating electrolyte migrating to a zone of the blue [PV] complex to evoke a demetallation and the formation of yellow free PV. A simple camera followed the color change. Our new evaluation method proved very efficient using the RGB channels of the images. With this, a calibration curve was obtained with a relatively wide linear range from 0.8 to 25 µM. Using the lower limit as an estimate for the LOD, it is clear that environmental monitoring is not yet possible with concentrations often below 1 µg L^*−*1^ (< 6 nM) [11]. Further optimization is required to preconcentrate GLY from an even larger reservoir or prolong the preconcentration times.

Our method seems promising to detect GLY in herbicide formulations and for visualizing spray drift, though the current detection limits are not yet adequate for surface water analysis. Isotachophoretic preconcentration provides the advantage of separating interfering compounds with different electrophoretic mobilities from GLY, such as glycine and certain carboxylic acids. Future research will explore potential matrix effects, including the influence of ionic strength and complexing agents like EDTA or humic acids, which may competitively complex copper ions. Additionally, the device can be adapted for other applications, such as analyzing GLY in (old) formulations in developing countries, where sales and applications are hardly regulated [5], or for detecting other ionic analytes and simplified copper analysis.

## Supplementary Information

Below is the link to the electronic supplementary material.

Supplementary file1 (MP4 14556 KB)

## References

[1] Benbrook CM. Trends in glyphosate herbicide use in the United States and globally. Environ Sci Eur. 2016;28(3):1. 10.1186/s12302-016-0070-0. 27752438 10.1186/s12302-016-0070-0 PMC5044953

[2] Huhn C. More and enhanced glyphosate analysis is needed. Anal Bioanal Chem. 2018;410(13):3041–5. 10.1007/s00216-018-1000-3. 29552731 10.1007/s00216-018-1000-3

[3] Bruggen AHCV, He MM, Shin K, Mai V, Jeong KC, Finckh MR, Morris JG. Environmental and health effects of the herbicide glyphosate. Sci Total Environ. 2018;616–617:255–68. 10.1016/j.scitotenv.2017.10.309. 29117584 10.1016/j.scitotenv.2017.10.309

[4] Gill JPK, Sethi N, Mohan A, Datta S, Girdhar M. Glyphosate toxicity for animals. Environ Chem Lett. 2017;16(2):401–26. 10.1007/s10311-017-0689-0.

[5] Oltramare C, Weiss FT, Staudacher P, Kibirango O, Atuhaire A, Stamm C. Pesticides monitoring in surface water of a subsistence agricultural catchment in Uganda using passive samplers. Environ Sci Pollut Res. 2022;30(4):10312–28. 10.1007/s11356-022-22717-2. 10.1007/s11356-022-22717-2 PMC9898397 36074287

[6] Okada E, Coggan T, Anumol T, Clarke B, Allinson G. A simple and rapid direct injection method for the determination of glyphosate and AMPA in environmental water samples. Anal Bioanal Chem. 2018;411(3):715–24. 10.1007/s00216-018-1490-z. 30535527 10.1007/s00216-018-1490-z

[7] Valle AL, Mello FCC, Alves-Balvedi RP, Rodrigues LP, Goulart LR. Glyphosate detection: methods, needs and challenges. Environ Chem Lett. 2018;17(1):291–317. 10.1007/s10311-018-0789-5.

[8] Wei P, Xiao L, Hou P, Wang Q, Wang P. A novel Cu(II)-assisted peptide fluorescent probe for highly sensitive detection of glyphosate in real samples: real application in test strips and smartphone. Anal Bioanal Chem. 2023;415(24):5985–96. 10.1007/s00216-023-04869-3. 37505235 10.1007/s00216-023-04869-3

[9] Yadav P, Zelder F. Detection of glyphosate with a copper(II)-pyrocatechol violet based GlyPKit. Anal Methods. 2021;13(38):4354–60. 10.1039/d1ay01168e. 34570143 10.1039/d1ay01168e PMC8498994

[10] Ulrich JC, Ferguson PL. Development of a sensitive direct injection LC-MS/MS method for the detection of glyphosate and aminomethylphosphonic acid (ampa) in hard waters. Anal Bioanal Chem. 2021;413:3763–74. 10.1007/s00216-021-03324-5. 33846826 10.1007/s00216-021-03324-5 PMC8154743

[11] Schwientek M, Rügner H, Haderlein S, Schulz W, Wimmer B, Engelbart L, et al. Glyphosate contamination in European rivers not from herbicide application? Water Res. 2024;263:122140. 10.1016/j.watres.2024.122140. 10.1016/j.watres.2024.122140 39096811

[12] Ferreira FT, Catalão KA, Mesquita RB, Rangel AO. New microfluidic paper-based analytical device for iron determination in urine samples. Anal Bioanal Chem. 2021;413:7463–72. 10.1007/s00216-021-03706-9. 34654951 10.1007/s00216-021-03706-9

[13] Zheng W, Wang K, Xu H, Zheng C, Cao B, Qin Q, Jin Q, Cui D. Strategies for the detection of target analytes using microfluidic paper-based analytical devices. Anal Bioanal Chem. 2021;413(9):2429–45. 10.1007/s00216-021-03213-x. 33712916 10.1007/s00216-021-03213-x

[14] Nery EW, Kubota LT. Sensing approaches on paper-based devices: a review. Anal Bioanal Chem. 2013;405(24):7573–95. 10.1007/s00216-013-6911-4. 23604524 10.1007/s00216-013-6911-4

[15] Salentijn GI, Grajewski M, Verpoorte E. Reinventing (bio) chemical analysis with paper. Anal Chem. 2018;90(23):13815–25. 10.1021/acs.analchem.8b04825. 30452240 10.1021/acs.analchem.8b04825 PMC6282107

[16] Schaumburg F, Kler PA, Carrell CS, Berli CL, Henry CS. USB powered microfluidic paper-based analytical devices. Electrophoresis. 2020;41(7–8):562–9. 10.1002/elps.201900273. 31677285 10.1002/elps.201900273

[17] Schaumburg F, Vidocevich JP, Gerlero GS, Pujato N, Macagno J, Kler PA, Berli CLA. A free customizable tool for easy integration of microfluidics and smartphones. Sci Rep. 2022;12(1):8969. 10.1038/s41598-022-13099-z. 35624294 10.1038/s41598-022-13099-z PMC9142529

[18] Niu J-C, Zhou T, Niu L-L, Xie Z-S, Fang F, Yang F-Q, Wu Z-Y. Simultaneous pre-concentration and separation on simple paper-based analytical device for protein analysis. Anal Bioanal Chem. 2018;410(6):1689–95. 10.1007/s00216-017-0809-5. 29327112 10.1007/s00216-017-0809-5

[19] Pagaduan JV, Sahore V, Woolley AT. Applications of microfluidics and microchip electrophoresis for potential clinical biomarker analysis. Anal Bioanal Chem. 2015;407(23):6911–22. 10.1007/s00216-015-8622-5. 25855148 10.1007/s00216-015-8622-5 PMC4725052

[20] Moghadam BY, Connelly KT, Posner JD. Isotachophoretic preconcentration on paper-based microfluidic devices. Anal Chem. 2014;86(12):5829–37. 10.1021/ac500780w. 24824151 10.1021/ac500780w

[21] Ramachandran A, Santiago JG. Isotachophoresis: Theory and microfluidic applications. Chem Rev. 2022;122(15):12904–76. 10.1021/acs.chemrev.1c00640. 35732018 10.1021/acs.chemrev.1c00640 PMC9373989

[22] Malá Z, Gebauer P. Recent progress in analytical capillary isotachophoresis. Electrophoresis. 2018;40(1):55–64. 10.1002/elps.201800239. 30039625 10.1002/elps.201800239

[23] Malá Z, Gebauer P. Analytical isotachophoresis 1967–2022: From standard analytical technique to universal on-line concentration tool. TrAC Trends Anal Chem. 2023;158:116837. 10.1016/j.trac.2022.116837.

[24] Daniele P. Copper(II) complexes of N-(phosphonomethyl)glycine in aqueous solution: a thermodynamic and spectrophotometric study. Talanta. 1997;45(2):425–31. 10.1016/S0039-9140(97)00156-2. 18967022 10.1016/s0039-9140(97)00156-2

[25] Madsen HEL, Christensen HH, Gottlieb-Petersen C, Andresen AF, Smidsrød O, Pontchour C-O, Phavanantha P, Pramatus S, Cyvin BN, Cyvin SJ. Stability constants of copper(II), zinc, manganese(ii), calcium, and magnesium complexes of N-(phosphonomethyl)glycine (glyphosate). Acta Chemica Scandinavica. 1978;32a:79–83. 10.3891/acta.chem.scand.32a-0079.

[26] Popov K, Rönkkömäki H, Lajunen LHJ. Critical evaluation of stability constants of phosphonic acids (IUPAC Technical Report). Pure Appl Chem. 2001;73(10):1641–77. 10.1351/pac200173101641.

[27] Kocyła A, Pomorski A, Kreżel A. Molar absorption coefficients and stability constants of Zincon metal complexes for determination of metal ions and bioinorganic applications. J Inorg Biochem. 2017;176:53–65. 10.1016/j.jinorgbio.2017.08.006. 28863280 10.1016/j.jinorgbio.2017.08.006

[28] Gerlero GS, MárquezDamián S, Kler PA. electromicrotransport v2107: Open-source toolbox for paper-based electromigrative separations. Comp Physics Commun. 2021;269:108143. 10.1016/j.cpc.2021.108143.

[29] Franck N, Berli CLA, Kler PA, Urteaga R. Multiphysics approach for fluid and charge transport in paper-based microfluidics. Microfluidics and Nanofluidics. 2022;26(11):87. 10.1007/s10404-022-02590-8.

[30] Graf HG, Biebl SM, Müller L, Breitenstein C, Huhn C. Capillary electrophoresis applied for the determination of acidity constants and limiting electrophoretic mobilities of ionizable herbicides including glyphosate and its metabolites and for their simultaneous separation. J Sep Sci. 2022;45(5):1128–39. 10.1002/jssc.202100952. 34984811 10.1002/jssc.202100952

[31] Gaš B. Peakmaster and Simul – Software tools for mastering electrophoresis. TrAC Trends Anal Chem. 2023;165:117134. 10.1016/j.trac.2023.117134.

[32] Schaumburg F, Berli CL. Assessing the rapid flow in multilayer paper-based microfluidic devices. Microfluid Nanofluid. 2019;23(8):1–10. 10.1007/s10404-019-2265-3.

[33] Sokołowska M, Bal W. Cu(II) complexation by “non-coordinating” N-2- hydroxyethylpiperazine-N-2-ethanesulfonic acid (HEPES buffer). J Inorg Biochem. 2005;99(8):1653–60. 10.1016/j.jinorgbio.2005.05.007. 15993944 10.1016/j.jinorgbio.2005.05.007

[34] Ewing MA, Robson AD. The use of MES buffer in early nodulation studies with annual Medicago species. Plant Soil. 1991;131(2):199–206. 10.1007/bf00009449.

[35] AguiarFilho SQ, Costa AMF, Santos Pereira AK, Cavallini GS, Pereira DH. Interaction of glyphosate in matrices of cellulose and diethylaminoethyl cellulose biopolymers: theoretical viewpoint of the adsorption process. J Mol Modeling. 2021;27(9):272. 10.1007/s00894-021-04894-y. 10.1007/s00894-021-04894-y 34468918

[36] Wimmer B. Alternative Wege in der Glyphosatanalytik. GIT LaborFachzeitschrift. 2022;4(66):28.

[37] Franck N, Schaumburg F, Kler PA, Urteaga R. Precise electroosmotic flow measurements on paper substrates. Electrophoresis. 2021;42(7–8):975–82. 10.1002/elps.202000271. 33433920 10.1002/elps.202000271

[38] Franck N, Vera Candioti L, Gerlero GS, Urteaga R, Kler PA. A simple method for the assessment of electrophoretic mobility in porous media. Electrophoresis. 2023;45(7–8):589–98. 10.1002/elps.202300180. 37853649 10.1002/elps.202300180

[39] Damián SM, Schaumburg F, Kler PA. Open-source toolbox for electromigrative separations. Comput Phys Commun. 2019;237:244–52. 10.1016/j.cpc.2018.11.015.

[40] Kaneta T, Ueda T, Hata K, Imasaka T. Suppression of electroosmotic flow and its application to determination of electrophoretic mobilities in a poly(vinylpyrrolidone)-coated capillary. J Chromatogr A. 2006;1106(1–2):52–5. 10.1016/j.chroma.2005.08.062. 16443452 10.1016/j.chroma.2005.08.062

[41] Wimmer B, Neidhardt H, Schwientek M, Haderlein SB, Huhn C. Phosphate addition enhances alkaline extraction of glyphosate from highly sorptive soils and aquatic sediments. Pest Manag Sci. 2022;78(6):2550–9. 10.1002/ps.6883. 35322519 10.1002/ps.6883

